# Chronic Activation of Corticotropin-Releasing Factor Type 2 Receptors Reveals a Key Role for 5-HT1A Receptor Responsiveness in Mediating Behavioral and Serotonergic Responses to Stressful Challenge

**DOI:** 10.1016/j.biopsych.2012.05.005

**Published:** 2012-09-15

**Authors:** Adi Neufeld-Cohen, Paul A.T. Kelly, Evan D. Paul, Roderick N. Carter, Elizabeth Skinner, Henry J. Olverman, Joan M. Vaughan, Orna Issler, Yael Kuperman, Christopher A. Lowry, Wylie W. Vale, Jonathan R. Seckl, Alon Chen, Pauline M. Jamieson

**Affiliations:** a Department of Neurobiology, Weizmann Institute of Science, Rehovot, Israel; b Centre for Cognitive and Neural Systems, University of Edinburgh, Edinburgh, United Kingdom; c Department of Integrative Physiology and Center for Neuroscience, University of Colorado Boulder, Boulder, Colorado; d Centre for Cardiovascular Science, Queens Medical Research Institute, University of Edinburgh, Edinburgh, United Kingdom; e The Clayton Foundation Laboratories for Peptide Biology, Salk Institute for Biological Studies, La Jolla, California

**Keywords:** Anxiety, corticotropin-releasing factor type 2 receptor, dorsal raphé nucleus, 5-HT type 1A receptor, serotonin, stress

## Abstract

**Background:**

The corticotropin-releasing factor type 2 receptor (CRFR2) is suggested to play an important role in aiding recovery from acute stress, but any chronic effects of CRFR2 activation are unknown. CRFR2 in the midbrain raphé nuclei modulate serotonergic activity of this key source of serotonin (5-HT) forebrain innervation.

**Methods:**

Transgenic mice overexpressing the highly specific CRFR2 ligand urocortin 3 (UCN3OE) were analyzed for stress-related behaviors and hypothalamic-pituitary-adrenal axis responses. Responses to 5-HT receptor agonist challenge were assessed by local cerebral glucose utilization, while 5-HT and 5-hydroxyindoleacetic acid content were quantified in limbic brain regions.

**Results:**

Mice overexpressing urocortin 3 exhibited increased stress-related behaviors under basal conditions and impaired retention of spatial memory compared with control mice. Following acute stress, unlike control mice, they exhibited no further increase in these stress-related behaviors and showed an attenuated adrenocorticotropic hormone response. 5-HT and 5-hydroxyindoleacetic acid content of limbic nuclei were differentially regulated by stress in UCN3OE mice as compared with control mice. Responses to 5-HT type 1A receptor challenge were significantly and specifically reduced in UCN3OE mice. The distribution pattern of local cerebral glucose utilization and 5-HT type 1A receptor messenger RNA expression levels suggested this effect was mediated in the raphé nuclei.

**Conclusions:**

Chronic activation of CRFR2 promotes an anxiety-like state, yet with attenuated behavioral and hypothalamic-pituitary-adrenal axis responses to stress. This is reminiscent of stress-related atypical psychiatric syndromes such as posttraumatic stress disorder, chronic fatigue, and chronic pain states. This new understanding indicates CRFR2 antagonism as a potential novel therapeutic target for such disorders.

Corticotropin-releasing factor (CRF) plays a fundamental role in regulating the behavioral and neuroendocrine responses to stressors (1). These effects, mediated via stimulation of the CRF type 1 receptor (CRFR1) include hypothalamic-pituitary-adrenal (HPA) axis activation and promotion of anxiety (2). In contrast, urocortins (Ucns) are the endogenous ligands for the CRF type 2 receptor (CRFR2) (3–7), which is suggested to modulate these stress responses.

Affective disorders are associated with CRF hyperactivity (8). CRF and Ucns alter the neurotransmitter systems targeted by antidepressants, in particular the serotonergic system (9,10). CRFR2 is abundant in the midbrain raphé nuclei (11–13), the main source of serotonin (5-HT) innervation to the forebrain. CRFR2 activity increases 5-HT neuronal firing rates and 5-HT release in efferent stress-related nuclei (14–18). This interaction may provide the major link between Ucns/CRFR2 and their effects on stress responses.

While acute pharmacologic stimulation of CRFR2 activates the HPA axis (19,20), both anxiogenic and anxiolytic effects on behavior are reported in rodents (21). Differing experimental paradigms or nonspecificity of the pharmacologic tools employed may be responsible (6,21). Urocortin 3 (Ucn3), however, is a highly specific agonist for CRFR2 (3,6,7). Close anatomical association between major Ucn3 terminal fields and CRFR2 in the limbic system and hypothalamus indicate this peptide is well placed to be an endogenous modulator of CRFR2 activity (22). Ucn3 appears to be anxiolytic when administered in acute experiments (21), but elevated Ucn3 in the perifornical hypothalamus is anxiogenic over a period of days (23). Although long-term CRFR2 stimulation is pertinent to human affective disorders, the effects of chronic CRFR2 activation on stress-related behaviors, the HPA axis, and 5-HT function are largely unknown. We have investigated this key issue by exploiting transgenic mice overexpressing Ucn3 (UCN3OE) widely throughout the brain.

## Methods and Materials

### Animals

Mice overexpressing Ucn3 were generated by pronuclear DNA microinjection of Ucn3 complementary DNA under the control of the ROSA26 promoter into C57BL/6 × BALB/c first generation oocytes, as previously described (24). Mice were housed in temperature and lighting controlled rooms (lights on 12 hours) with free access to laboratory chow and water. All experimental mice were the offspring of a sire heterozygous for the transgene and a transgene-negative dam from the closed colony. Thus, UCN3OE mice were heterozygous for the transgene, and control mice were transgene-negative littermates. Experiments were carried out on male mice between 10 and 14 weeks old. Principles of Laboratory Animal Care (National Institutes of Health Publication No. 85–23, 1985) were followed. All procedures were approved by The Salk Institute Animal Use and Care Committee, The Weizmann Institute Animal Use and Care Committee, or the United Kingdom Animals (Scientific Procedures) Act, 1986.

### Behavioral Testing

All tests were carried out in group-housed mice during the dark phase of the light cycle. Mice were habituated in the home cage in a dark room for 2 hours before testing with constant background white noise (52 dB). For acute restraint stress, mice were subjected to 30 minutes in a ventilated 50 mL plastic centrifuge tube and returned to the home cage for 30 minutes before testing. Separate cohorts of animals were used for 1) rotarod and behavioral testing under basal conditions, *n* = 14 to 16; 2) behavioral testing following restraint stress, *n* = 11 to 15; and 3) Barnes maze analysis, *n* = 13 to 14. Order of testing was rotarod test (cohort 1 only), open-field test, elevated plus-maze (EPM), light-dark transfer test (LDT), and tail suspension test (TST), with 48 to 72 hours between tests. These animals were not further used for other experiments described in this study.

### Rotarod Test

Mice were placed on a standard rotarod apparatus with the speed increasing linearly from 5 to 70 rpm over a 5-minute period. Latency to fall from the rotating drum was recorded. For evaluation of motor skill learning, mice were tested three times in quick succession, and this was repeated three times over a period of 4 hours. To test motor memory, another three-run cycle was performed the following day.

### Open-Field Test

The apparatus and experimental conditions were as previously described (25). Each mouse was placed in the center of the apparatus to initiate a 10-minute test session. Time spent in the inner squares of the arena and the total number of squares crossed were quantified.

### Elevated Plus-Maze

The apparatus and experimental conditions were as previously described (26). Number of open-arm and closed-arm entries and time spent on the open or closed arms were scored. Arm entries were defined as entry of all four paws into the arm. Total arm entries were taken as an index of locomotor activity.

### Light/Dark Transfer Test

The apparatus and experimental conditions were as previously described (25). During a 5-minute test session, the latency to enter the light compartment and the number of entries and time spent in the light compartment were measured.

### Tail Suspension Test

Mice were suspended from a metal horizontal rod by taping by the base of the tail for 6 minutes. The duration of immobility was scored. Any mouse climbing onto the rod was excluded from analysis.

### Barnes Maze

The apparatus consists of a 90 cm diameter Plexiglas circular platform, with 20 holes (5 cm diameter) equidistant around the periphery and surrounded by visual cues. One hole leads to an escape chamber, always located underneath the same randomly determined hole for each mouse. The initial training session was performed by placing the mouse in the escape box for 1 minute and the first trial started 1 minute later. Mice were placed in the middle of the platform under a cup, which was removed to initiate each trial. These were carried out with 80 dB white noise and 400 lux lighting as aversive stimuli. The trial ended upon entry to the escape chamber or after 5 minutes had elapsed. Sound and light were turned off immediately upon successful termination of the trial and the mouse was allowed to remain in the dark for 1 minute. Each mouse was subject to 16 trials over a period of 10 days. First error was scored as the number of holes away from the correct hole first approached by the mouse. An error was defined as searching any hole that did not have the escape chamber beneath it. Time to complete the trial and the search strategy were also recorded: random, unsystematic hole searches with crossings through the maze center; serial, systematic hole searches (every hole or every other hole) in a clockwise or counterclockwise direction; and spatial, moving directly to the target hole or to an adjacent hole before visiting the target. A probe test in which the escape chamber was closed off was run on the final day. For scoring, the maze was divided into four quadrants, with the target hole in the center of the periphery of one quadrant. Latency to first approach the target hole and time spent, crossings into, and the distance travelled in the correct quadrant were scored.

### Hypothalamic-Pituitary-Adrenal Axis Activity

Blood samples were collected from individually housed mice by retro-orbital eye bleed from unanesthetized animals within 15 seconds of disturbance of the home cage. For basal HPA axis activity, samples were collected at 7:00 am and 5:00 pm (lights on 6:00 am). For HPA axis response to stress, samples were collected after 2 minutes and 10 minutes of restraint stress at 7:00 am. Individual animals were sampled only once (*n* = 13–15). Adrenocorticotropic hormone (ACTH) and corticosterone levels were measured in duplicate in unextracted plasma samples using commercially available radioimmunoassay kits as previously described (19). Adrenal glands were weighed following dissection from a separate cohort of animals.

### Ucn3 Radioimmunoassay

Ucn3 was measured in whole brain (*n* = 3) by in-house radioimmunoassay as previously described (27).

### Messenger RNA Analysis by In Situ Hybridization Histochemistry and Quantitative Real-Time Polymerase Chain Reaction

Antisense and sense (control) RNA probes were generated using mouse Ucn3 complementary DNA and labeled with DIG-11-UTP using a labeling kit from Roche Molecular Biochemicals (Burgess Hill, United Kingdom). In situ hybridization for Ucn3 messenger RNA (mRNA) (*n* = 3) and quantitative polymerase chain reaction (qPCR) for CRFR1, CRFR2, and CRF mRNA expression (*n* = 4−7) were carried out as previously reported (28,29). Primers for serotonin 1A receptor (5-HT1AR) (*Htr1a*) qPCR: 5'-GTGCACCATCAGCAAGGACC-3' and 5'-GCGCCGAAAGTGGAGTAGAT-3' corresponded to nucleotides 1648-1667 and 1698−1717, respectively. Primers for serotonin reuptake transporter (*Slc6a4*) qPCR: 5'-GGGTTTGGATAGTACGTTCGCA-3' and 5'-CATACGCCCCTCCTGATGTC-3' corresponded to nucleotides 1490-1511 and 1650-1669, respectively (*n* = 3−7).

### Local Cerebral Glucose Utilization

Local cerebral glucose utilization (LCMRglc) was determined using a 2-deoxyglucose autoradiographic imaging protocol modified from the original technique (30) as described by us previously (31). Equal numbers (*n* = 7) of animals from each genotype were randomly allocated to a drug treatment group and injected (intraperitoneal) with either 10 mg.kg^−1^ 8-hydroxy-N,N-dipropyl-2-aminotetralin (8-OH-DPAT), 25 mg.kg^−1^ 1-(2,5-dimethoxy-4-iodophenyl)-2-aminopropane (DOI), or vehicle (.1 mL .9% sodium chloride). Ten minutes after injection of 8-OH-DPAT (or vehicle) or 20 minutes after injection of DOI (or vehicle) measurement of LCMRglc was initiated by injection (intraperitoneal) of 5 μCi [^14^C]-2-deoxyglucose in .4 mL .9% sodium chloride. After 45 minutes, mice were decapitated and the brains processed for quantitative autoradiographic imaging. Analysis of autoradiograms was performed as described previously (32,33).

### High-Performance Liquid Chromatography Analysis of Tissue Concentrations of 5-HT and 5-Hydroxyindoleacetic Acid

Mice (*n* = 8) were killed by decapitation under basal conditions or 24 hours following restraint stress. Brains were stored at −80°C until analysis. Areas selected for microdissection (Table S1 in Supplement 1) were identified by comparisons with a standard mouse brain stereotaxic atlas (34) and included the raphé nuclei, an anxiety-related raphé-amygdala-subiculum circuit, and septal areas implicated in stress recovery. High-performance liquid chromatography analysis of 5-HT and 5-hydroxyindoleacetic acid (5-HIAA) were performed as previously described (35).

### Statistical Analyses

Statistical analyses employed two-way analysis of variance with post hoc analysis using Fisher's protected least significant difference test or the two-tailed Student *t* test, as appropriate. Data are presented as mean ± SEM. Differences were considered statistically significant at *p* < .05.

## Results

### Brain Overexpression of Ucn3 in UCN3OE Mice

**Figure 1:**
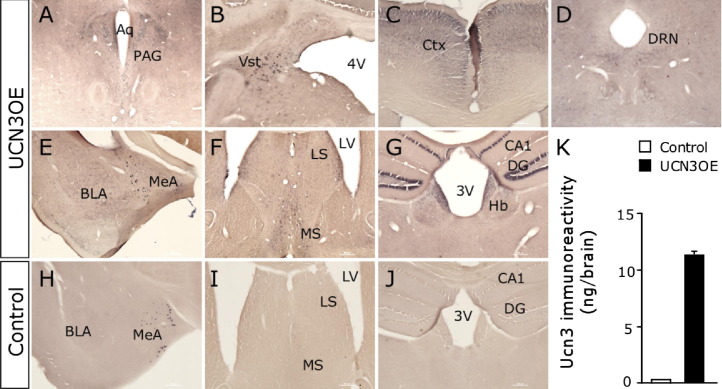
Mice overexpressing urocortin 3 (UCN3OE) express Ucn3 in normal and ectopic brain nuclei. In situ hybridization for Ucn3 messenger RNA in UCN3OE **(A–G)** and control **(H–J)** mice showed an endogenous pattern of expression in both control and UCN3OE mice in the MeA **(E)**. Ectopic Ucn3 messenger RNA expression was present in the PAG **(A),** Vst **(B),** Ctx **(C),** DRN **(D)**, BLA **(E)**, LS and MS nuclei **(F)**, and the hippocampal CA1, DG, and Hb **(G)** of UCN3OE mice. **(J)** Ucn3 peptide levels were increased in UCN3OE brains **(K)**. *n* = 3. 3V, third ventricle; 4V, fourth ventricle; Aq, cerebral aqueduct; BLA, basolateral amygdala; CA, cornu ammonis; Ctx, cortex; DG, dentate gyrus; DRN, dorsal raphé nucleus; Hb, habenula; LS, lateral septal; LV, lateral ventricle; MeA, medial amygdala; MS, medial septal; PAG, periaqueductal gray; Ucn3, urocortin 3; Vst, vestibular nucleus.

Control brains showed endogenous Ucn3 mRNA expression in the medial amygdala, perifornical area, and the bed nucleus of the stria terminalis as previously reported (6,22). In situ hybridization demonstrated widespread overexpression of Ucn3 mRNA in UCN3OE brains (Figure 1A–G), while increased peptide levels were confirmed by radioimmunoassay (Figure 1K). In addition to expressing Ucn3 mRNA in these endogenous sites, UCN3OE mice have ectopic expression in numerous brain nuclei, including the basolateral amygdala (BLA); cornu ammonis (CA)1, CA3, subiculum, and dentate gyrus of the hippocampus; lateral septum (LS) and medial septum; lateral and medial habenula; caudate putamen; piriform cortex; arcuate nucleus and ventromedial hypothalamus; vestibular nucleus; dorsal raphé nucleus (DRN); periaqueductal gray; and cortex. Several of these brain regions, including the LS, ventromedial hypothalamus, and DRN, express high levels of CRFR2 (12). CRF, CRFR1, and CRFR2 mRNA levels were quantified in their principle sites of expression within anxiety-related circuits (Table S2 in Supplement 1). Ucn3 overexpression did not significantly alter their expression in any area.

### UCN3OE Mice Show Increased Stress-Related Behaviors

**Figure 2:**
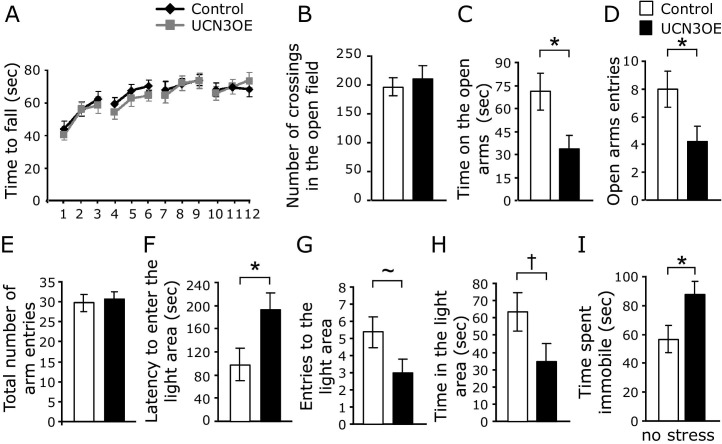
Mice overexpressing Ucn3 (UCN3OE) show higher anxiety-like behavior under basal conditions. **(A)** Motor performance in the rotarod test and **(B)** locomotor activity in the open field test. UCN3OE mice **(C)** spent less time on and **(D)** made fewer entries into the open arms of the elevated plus-maze but did not differ in **(E)** the total number of arm entries made compared with control mice. In the light/dark transfer test, UCN3OE mice showed a **(F)** longer latency to enter the light compartment and a strong tendency to **(G)** less entries into the light and **(H)** less time in the light. **(I)** UCN3OE mice spent more time immobile in the tail suspension test. *p < .05, ∼*p* = .06, †*p* = .07. *n* = 14 to 16. Ucn3, urocortin 3.

Motor learning and memory were tested using a 2-day, 12-trial rotarod test. Mice overexpressing Ucn3 showed no motor deficits (Figure 2A). Similarly, in the open-field test, UCN3OE mice and littermate control mice showed no differences in total number of crossings, indicating similar locomotor behavior (Figure 2B).

Assessment of stress-related behaviors was initially carried out under basal conditions, without exposing mice to any stress other than that caused by the test itself. Mice overexpressing Ucn3 exhibited higher anxiety-like behavior, as evidenced by less time spent on the open arms (Figure 2C) and less entries made to the open arms (Figure 2D) of the EPM. Again, this was not due to differences in locomotion since the total number of entries was similar between groups (Figure 2E). In the LDT, UCN3OE mice showed increased anxiety-like behavior with a longer latency to enter the light compartment (Figure 2F) and a tendency toward less entries into the light (Figure 2G, *p* = .06) and less time spent in the light (Figure 2H, *p* = .07). Mice overexpressing Ucn3 also spent more time immobile than control mice in the TST (Figure 2I) (36).

**Figure 3:**
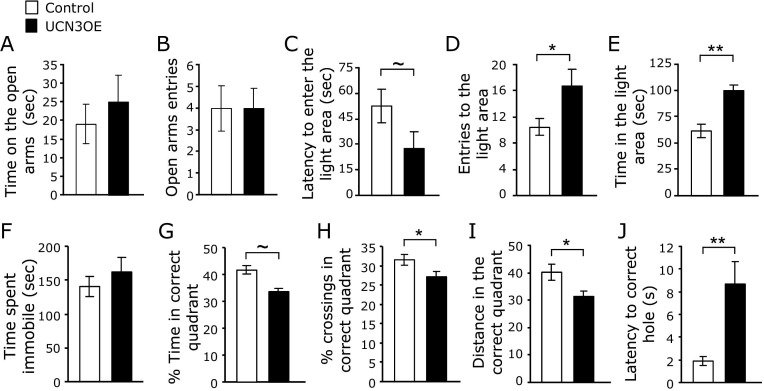
Mice overexpressing urocortin 3 (UCN3OE) show attenuated behavioral responses to acute restraint stress and spatial memory deficits. UCN3OE mice **(A)** spent similar time on and **(B)** made a similar number of entries into the open arms of the elevated plus-maze as control mice. UCN3OE mice showed a tendency to **(C)** a shorter latency to enter and **(D)** made significantly more entries into and **(E)** spent more time in the light compartment in the light/dark transfer test. **(F)** Immobility in the tail suspension test following stress. *n* = 11 to 15. **(G)** Time spent, **(H)** crossings in, and **(I)** distance travelled in the correct quadrant and **(J)** the latency to find the escape hole during the probe test in the Barnes maze, *n* = 13 to 14. *p < .05, **p < .01, ∼*p* = .09.

It has been previously shown that activation of CRFR2 affects anxiety-like behavior (23,37,38) under stressed conditions. Following exposure to acute restraint stress (separate cohort), there were no longer differences between behavior of UCN3OE mice and control mice in the EPM (Figure 3A, B; Figure S1 in Supplement 1). This appears due to a stress-related increase in anxiety-like behavior in control but not UCN3OE mice. In the LDT, UCN3OE mice entered the light compartment more often (Figure 3D) and spent significantly more time in the light (Figure 3E) than control mice. This appeared to be due to a decrease in anxiety-like behavior in UCN3OE mice. Stress increased immobility in the TST in both genotypes, but there was no difference between genotypes in immobility following stress (Figure 3F). Overall stress removed the affective behavioral differences between UCN3OE and control mice seen under basal conditions.

### Spatial Learning and Memory in the Barnes Maze

Both control and UCN3OE mice showed similar learning in the Barnes maze (39), as shown by measures of reduction in distance of the first hole checked from the escape hole (first error), reduction in number of errors made in each trial set, shorter latency to escape over 16 trials, and the strategy used to locate the escape hole (Figure S1 in Supplement 1). Retention of learning was tested using a single trial probe test. Mice overexpressing Ucn3 explored the correct quadrant significantly less than control mice, as shown by the number of crossings (Figure 3H) and distance travelled (Figure 3I) in the correct quadrant. In addition, UCN3OE mice showed a longer latency to approach the escape hole (Figure 3J). Hence, UCN3OE mice show impaired spatial memory retention.

### HPA Axis Responsiveness

**Figure 4:**
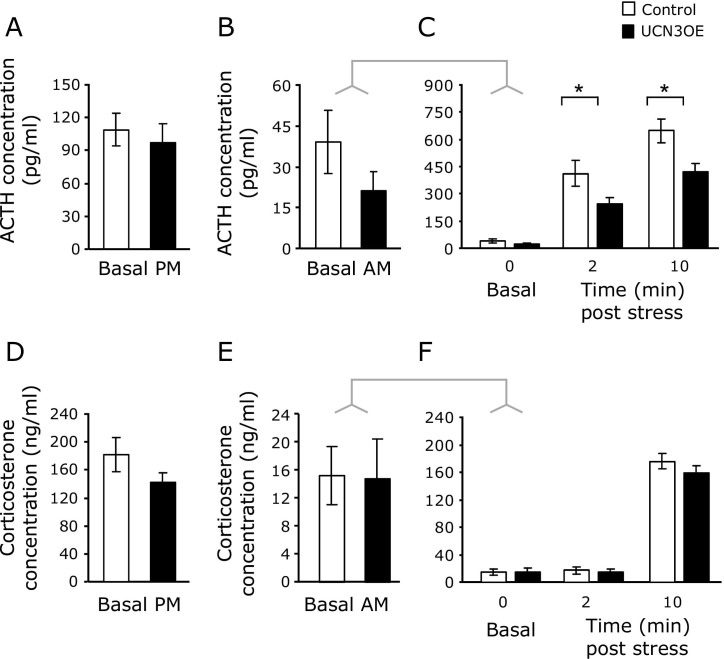
Mice overexpressing Ucn3 (UCN3OE) and control mice show similar basal hypothalamic-pituitary-adrenal axis activity but an attenuated adrenocorticotropic hormone (ACTH) response upon acute stress. Plasma ACTH levels were measured in the **(A)** evening and **(B)** morning under basal conditions. **(C)** On acute stress, UCN3OE mice show attenuated ACTH levels. No differences were found in plasma corticosterone levels in the **(D)** evening and **(E)** morning or **(F)** following stress. *n* = 13 to 15. *p < .05.

ACTH and corticosterone levels measured under stress-free basal conditions were not different between genotypes at the diurnal peak (Figure 4A, D) and trough (Figure 4B, E) of the circadian rhythm. Following restraint stress, UCN3OE mice showed a significantly attenuated rise in ACTH levels over 10 minutes (Figure 4C), although the rise in corticosterone levels over this time was similar to control mice (Figure 4F). Total adrenal weight did not differ from control mice (control mice 3.62 ± .27 mg, UCN3OE 3.69 ± .33 mg, *n* = 6–8).

### Serotonergic Function Is Altered in UCN3OE Mice

Following challenge with the 5-HT1AR-specific agonist 8-OH-DPAT, analysis of variance revealed a main effect of treatment in all brain areas examined and of genotype in the hippocampal CA3 [*F*(1,24) = 15.082, *p* < .01] and molecular layer [*F*(1,24) = 5.921, *p* < .05], prefrontal cortex [*F*(1,24) = 7.407, *p* < .05], dorsal subiculum [*F*(1,24) = 4.645, *p* < .05], and the medial septum [*F*(1,24) = 5.538, *p* < .05] (Table 1 and Table S3 in Supplement 1). There was a significant interaction of treatment × genotype in the hippocampal CA3 [*F*(1,24) = 11.081, *p* < .01], prefrontal cortex [*F*(1,24) = 7.407, *p* < .05], dorsal subiculum [*F*(1,24) = 8.258, *p* < .01], dentate gyrus [*F*(1,24) = 4.902, *p* < .05], and lateral striatum [*F*(1,24) = 4.558, *p* < .05]. In contrast, when challenged with the serotonin 2C receptor-specific agonist DOI, despite a main effect of treatment in many brain areas, ranging from hippocampal field CA3 [*F*(1,24) = 33.923] to parietal cortex [*F*(1,24) = 11.461], there was no main effect of genotype or any significant interaction of genotype with treatment on LCMRglc (Table 2 and Table S4 in Supplement 1), demonstrating a differential response to 8-OH-DPAT only between genotypes.

**Figure 5:**
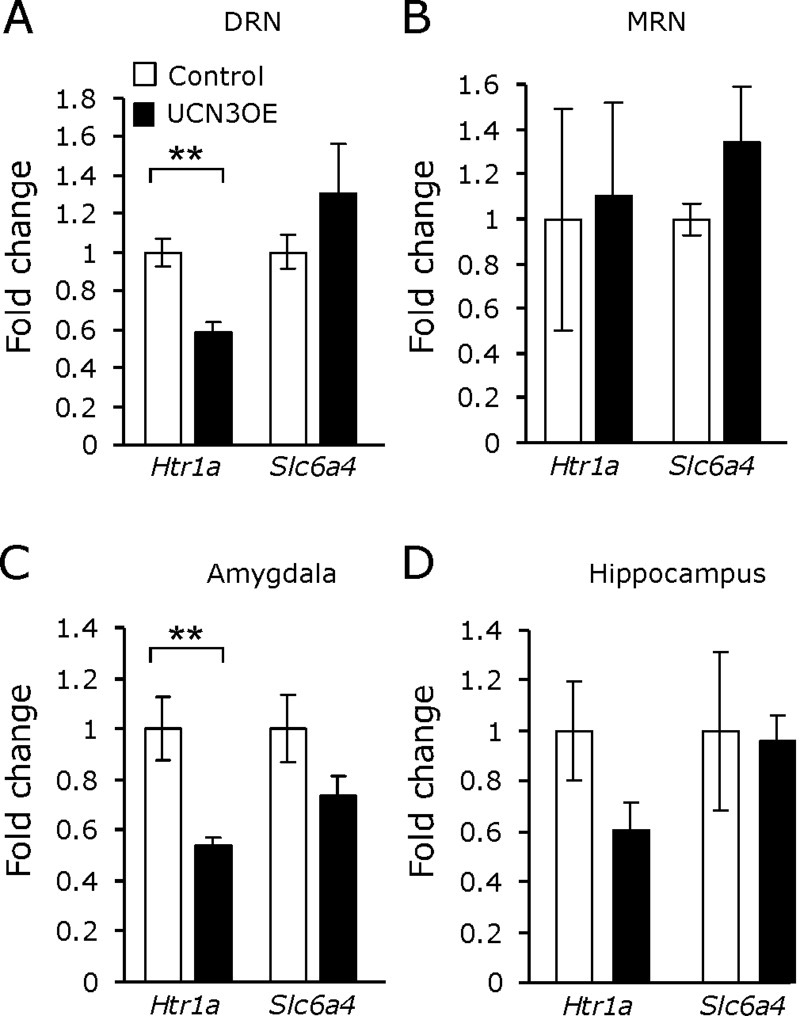
Mice overexpressing Ucn3 (UCN3OE) show decreased basal *htr1a* messenger RNA expression within the **(A)** dorsal raphé nucleus (DRN) and **(C)** amygdala but not **(B)** median raphé nucleus (MRN) or **(D)** hippocampus. Basal *htr1a* and *slc6a4* messenger RNA expression within stress-related brain regions in mice overexpressing urocortin 3 and control mice. Panels on the right depict the location of the tissue dissection. *n* = 4 to 7, except for MRN *n* = 3 to 4. **p < .01.

Further post hoc analysis (Tables 1 and 2) revealed no significant differences in LCMRglc between vehicle-treated control and UCN3OE mice, indicating no effect of genotype on constitutive cerebral glucose utilization, likely reflecting adaptation to lifelong altered function. Following 8-OH-DPAT, the decrease in LCMRglc was significantly less than in control mice, and, in fact, LCMRglc was not different from vehicle-treated animals in the majority of brain regions studied, indicating an attenuated response to 5-HT1AR stimulation. In contrast, when challenged with DOI, there was no significant difference in LCMRglc between the genotypes (Table 2). Messenger RNA expression of the 5-HT1AR was significantly decreased in the DRN and amygdala of the UCN3OE mice (Figure 5; Figure S2 in Supplement 1). Serotonin reuptake transporter mRNA expression did not differ between genotypes in any brain region examined.

**Figure 6:**
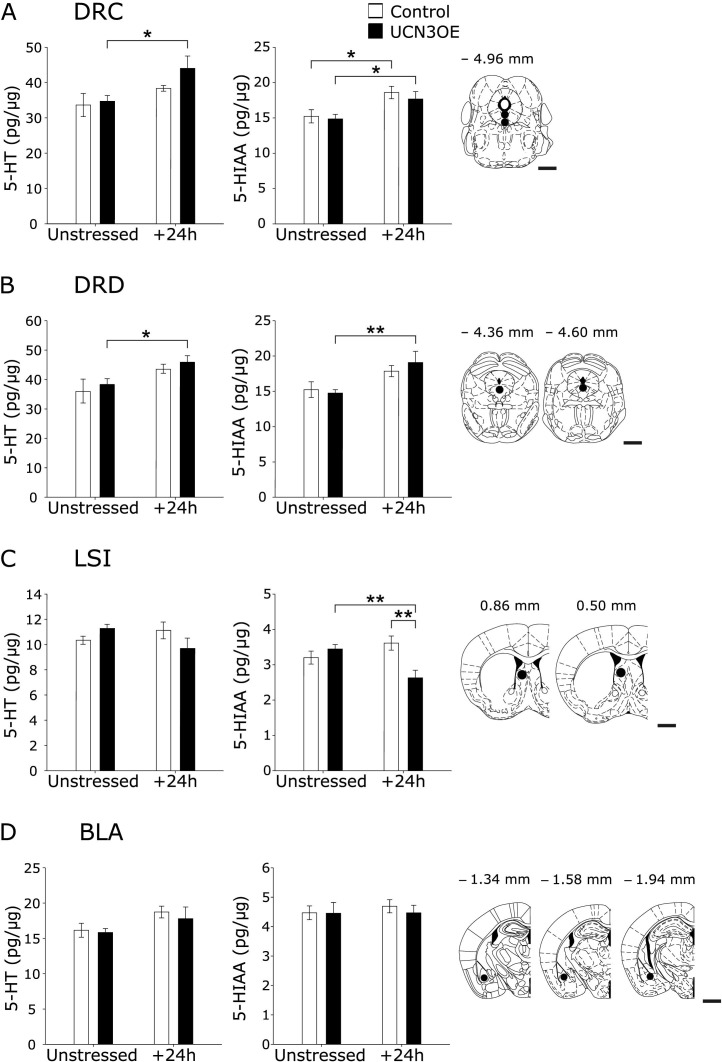
Mice overexpressing Ucn3 (UCN3OE) show stress-induced alterations in tissue concentration of serotonin (5-HT) and 5-hydroxyindoleacetic acid (5-HIAA) levels in stress-related brain regions. 5-HT (left panel) and 5-HIAA (middle panel) concentrations under unstressed and 24 hours poststress (+24h) conditions in the **(A)** dorsal raphé nucleus, caudal part (DRC), **(B)** dorsal raphé nucleus, dorsal part (DRD), **(C)** lateral septum, intermediate part (LSI), and **(D)** basolateral amygdala (BLA). Panels on the right depict the location of the microdissection and approximate distance from bregma (reprinted from Paxinos and Franklin [34] with permission from Elsevier, copyright 2001). *n* = 8. Scale bar = 1 mm. *p < .05; **p < .01. Ucn3, urocortin 3.

Consistent with the observation of unchanged constitutive brain function in UCN3OE mice, there was no difference in 5-HT and 5-HIAA content of selected brain nuclei when compared with control mice under basal conditions (Figure 6; Table S5 in Supplement 1). There were significant main effects of stress on 5-HT content in the caudal DRN [*F*(1,28) = 7.566, *p* < .05], dorsal DRN [*F*(1,28) = 8.477, *p* < .01], and the BLA [*F*(1,28) = 4.337, *p* < .05]. For 5-HIAA, there was a significant main effect of stress in the caudal DRN [*F*(1,28) = 13.278, *p* < .01] and dorsal DRN [*F*(1,28) = 10.495, *p* < .01]. There was a significant interaction (genotype × stress) on both 5-HT [*F*(1,28) = 4.225, *p* < .05] and 5-HIAA [*F*(1,28) = 5.371, *p* < .05] concentrations in the intermediate LS.

Post hoc analysis revealed that 24 hours following a stressor, there was an increase in 5-HIAA content of the caudal DRN (Figure 6A) of control mice. In contrast, UCN3OE mice showed a more robust serotonergic response with significant increases in 5-HT and 5-HIAA in both the caudal and dorsal DRN poststress (Figure 6A, B). They also showed decreased 5-HIAA, in the intermediate LS poststress (Figure 6C). There were no effects of stress or genotype in the central amygdala, medial septum, or subiculum (data not shown).

## Discussion

We show that specific CRFR2 activation by chronic Ucn3 overexpression increases baseline stress-related behaviors and yet attenuates the affective impacts of stress and aspects of HPA axis function. The underlying mechanism for this is plausibly reduced 5-HT1AR signaling in the raphé nuclei.

Ucn3 overexpression should result in continuous and chronic stimulation and relative hyperactivation of CRFR2. Ucn3 overexpression has potential to stimulate endogenous CRFR2 at the site of expression, e.g., in the LS, BLA, hippocampal CA1, and the DRN (12,13), or to exert effects via axonal transport of Ucn3 to CRFR2 fields, e.g., the habenula has no CRFR2 but provides projections to the DRN (40–42). Importantly, CRFR2 levels in functionally significant sites, including the paraventricular nucleus, LS, median raphé nucleus (MRN), and DRN, are not affected by the continuous overexpression of ligand, a finding in keeping with unaltered peripheral expression of CRFR2 in these mice (24).

Mice overexpressing Ucn3 display an increase in stress-related behaviors under basal circumstances, suggesting chronic CRFR2 activation promotes an anxiety-like state. This extends and accords with the previously observed increase in anxiety seen following viral overexpression of Ucn3 in hypothalamus (23). Anxiety impairs spatial memory (43–48), plausibly accounting for the reduced retention of the spatial memory seen in UCN3OE mice. Conversely, following a stressor, indices of anxiety-like behavior and immobility in the TST in UCN3OE mice were similar to or lower than in control mice. This could be due to a ceiling effect of the enhanced anxiety levels in the UCN3OE mice. However, UCN3OE mice even showed some anxiolysis under stressful conditions. Overall, CRFR2 hyperactivation in this model displays a dual and contrasting effect under basal and stress conditions, in agreement with the suggested roles of CRFR2 in contributing to the recovery phases of the stress response (49–51). These effects may be, at least in part, due to direct effects of CRFR2 stimulation by Ucn3 on behavior. However, current understanding of CRFR2 function in relation to this aspect has moved mechanistic theories away from the originally proposed direct opposition of CRFR1 action and toward more complex regulation of other neurotransmitter systems by CRFR2 activity, in particular 5-HT function (15,16,29,52). Consistent with this, 5-HT function is significantly altered in UCN3OE mice, with evidence of attenuated 5-HT1AR responsiveness.

The response to a 5-HT1AR agonist was altered in extrapyramidal brain areas lacking in 5-HT1AR (53,54) but which receive 5-HT projections from the DRN (55,56) in UCN3OE mice, inferring the effect is mediated in the DRN rather than in the forebrain structures themselves. Serotonin innervation of the forebrain is supplied by the MRN and DRN (57), both of which express CRFR2 (12), and it is likely the MRN may be similarly affected. However, 5-HT1AR mRNA expression and 5-HT and 5-HIAA content following stress exposure were altered only in the DRN of UCN3OE mice, suggesting this may be the main site where effects on 5-HT function by CRFR2 are mediated. In keeping with this, 5-HT1AR was downregulated in the UCN3OE amygdala, an area where 5-HT projections are supplied by the DRN (57).

Mice overexpressing Ucn3 show enhanced stress-induced 5-HT and 5-HIAA content in the DRN, consistent with their attenuated response to 5-HT1AR agonist and reduced 5-HT1AR expression here, as less autoinhibition by 5-HT1AR correlates with increased activation and firing rates of 5-HT neurons. While the dorsal DRN is the classic anxiety-related subregion of the DRN activated by anxiogenic drugs, social defeat, fear-potentiated startle, and urocortin 2 (15,58–60), the caudal DRN may mediate stress-induced alterations of 5-HT in forebrain regions including the LS (61). A differential genotype effect in the LS was also observed, with decreased 5-HT and 5-HIAA levels in UCN3OE mice poststress. This area not only receives both DRN and MRN projections but is also implicated in promoting stress-related behaviors mediated by direct activation of the CRFR2 expressed here (62,63).

Corticotropin-releasing factor receptor stimulation in the raphé nuclei regulates efferent 5-HT release in a site-specific manner with increases in cortex and hippocampus but decreases in other regions including the LS and striatum (10). This is presumed due to differential CRFR1 versus CRFR2 activation (9). Our results implicate CRFR2 in modulating these effects following a stressor. In contrast, no difference in the 5-HT responses of UCN3OE mice in amygdala or subiculum compared with control mice indicates this anxiety-related circuit may not be key to their stress-related phenotype or, given the well-documented effects of stress here, that 24 hours is not the crucial time point to observe any differential effects.

Dysregulated 5-HT functioning is recognized to underlie the pathophysiology of stress-related psychopathologies, including anxiety disorders and depression, with decreased 5-HT1AR activity associated with these disorders (64–66). Learned helplessness, a behavioral model for anxiety and affective disorders, is dependent on 5-HT activity in the DRN and is proposed to result from hyperactivation of 5-HT neurons in the DRN during exposure to uncontrollable stress (67), leading to internalization of inhibitory serotonin 1A autoreceptors, thus sensitizing other DRN 5-HT neurons to subsequent stress (68,69). In addition to modulating 5-HT neuronal activity, CRFR2 stimulation mediates the behavioral aspects of this process (70). Thus, we hypothesize the phenotype of the UCN3OE mice is a consequence of the continued activation of CRFR2 resulting in a similar process. We propose a model where in the healthy animal, Ucn3 is rapidly released in response to an acute stressor (19,71) and is an important mediator of the stress recovery process (51). However, in UCN3OE mice, ongoing Ucn3 stimulation of CRFR2 models a chronically stressed animal with increased anxiety-like behaviors and decreased responsiveness of 5-HT1AR autoreceptors.

Contrary to what might be anticipated given their behavioral phenotype, UCN3OE mice showed no differences in ACTH or corticosterone levels under basal conditions but exhibited attenuated HPA axis activation (ACTH) in response to stress. Adrenal weight and corticosterone response to stress were not different between genotypes, suggesting possibly greater adrenocortical responses to ACTH in UCN3OE mice, but more detailed analyses over a longer time course are required to draw any definite conclusions.

Blunting of ACTH responses is observed in a subgroup of affective disorders, including atypical depression, posttraumatic stress disorder, and in adults following childhood abuse (72–74), and HPA axis hyporeactivity has been reported in rodents exposed to maternal separation (75–77). Proposed mechanisms include desensitization of CRF receptors and hence the HPA axis by chronically elevated ligand (78) or hypoactivity of CRF neurons based on findings of low CRF in cerebrospinal fluid in patients in certain disorders including chronic fatigue and atypical depression (79–82). In either case, this would appear to be a maladaptation to chronic stress exposure.

Therefore, while attenuated HPA axis and behavioral responses to stress may well reflect the proposed contrasting role of CRFR2 under basal and stressed conditions (49,50), they may also represent maladaptation of the stress response, akin to that observed in illnesses such as atypical depression and posttraumatic stress disorder. Atypical depression has been reported to have a higher comorbidity of anxiety disorders than other subtypes of depression (83,84). Furthermore, gender differences in clinical presentation, with women reported to show more atypical and anxiety symptoms than men (85–87), have led to suggestions that similar serotonergic dysfunctioning underpins both atypical depression and anxiety disorders and that selective serotonin reuptake inhibitors may be more effective than other antidepressant medications in women. Our findings in UCN3OE mice provide further evidence for this link between anxiety, hyporesponsiveness of the HPA axis, and associated dysregulation of serotonergic function, specifically attenuated 5-HT1AR responsiveness. Thus, the role of CRFR2 and urocortins in relation to these psychiatric conditions appears worthy of further study. There is much to be elucidated regarding the complex regulation of serotonergic function by CRFR2, and UCN3OE mice may provide a useful model in this respect.

## Figures and Tables

**Table 1:** LCMRglc in Brain Regions of Control and UCN3OE Mice in Response to 5-HT1AR Agonist 8-OH-DPAT

	Control			UCN3OE		
	Saline	8-OH-DPAT	%	Saline	8-OH-DPAT	%
Dorsal Raphé Nucleus	35 ± 3	25 ± 3^a^	−29	33 ± 3	18 ± 5^a^	−46
Median Raphé Nucleus	46 ± 2	36 ± 3^a^	−22	45 ± 3	24 ± 7^a^	−47
Neocortex						
Orbitofrontal	62 ± 4	39 ± 4^a^	−37	62 ± 5	52 ± 4	−16
Frontal	46 ± 3	33 ± 3^a^	−28	48 ± 4	42 ± 3	−12
Anterior cingulate	48 ± 4	31 ± 4^a^	−36	45 ± 5	39 ± 4	−13
Prefrontal^b^^,^^c^	47 ± 5	22 ± 3^a^	−53	47 ± 4	42 ± 2	−11
Somatosensory	54 ± 4	38 ± 5^a^	−29	52 ± 5	45 ± 3	−13
Parietal	54 ± 4	34 ± 4^a^	−35	48 ± 5	43 ± 3	−10
Posterior cingulate	51 ± 4	32 ± 3^a^	−37	49 ± 5	42 ± 2	−15
Piriform	39 ± 3	24 ± 2^a^	−38	41 ± 3	35 ± 5	−14
Entorhinal	35 ± 3	24 ± 3^a^	−32	33 ± 2	29 ± 2	−12
Hippocampus						
Molecular layer^b^	41 ± 4	26 ± 3^a^	−38	44 ± 3	38 ± 2	−14
Dorsal subiculum^b^^,^^c^	38 ± 3	24 ± 3^a^	−39	36 ± 3	32 ± 2	−11
Dentate gyrus^c^	25 ± 1	15 ± 1^a^	−38	23 ± 2	20 ± 1	−13
Dorsal CA1	35 ± 3	23 ± 4^a^	−36	33 ± 2	28 ± 3	−15
CA2	33 ± 3	21 ± 2^a^	−36	34 ± 3	30 ± 4	−12
Ventral CA1	33 ± 2	20 ± 2^a^	−39	31 ± 3	27 ± 2	−13
Ventral subiculum	29 ± 2	20 ± 2^a^	−31	28 ± 3	25 ± 3	−11
CA3	33 ± 2	16 ± 2^a^	−52	34 ± 2	29 ± 1	−15
Extrapyramidal Areas						
Medial striatum	43 ± 3	33 ± 3^a^	−24	44 ± 3	39 ± 2	−11
Lateral striatum^c^	48 ± 4	29 ± 3^a^	−39	46 ± 3	41 ± 3	−11
Globus pallidus	32 ± 3	22 ± 1^a^	−31	33 ± 2	29 ± 2	−12
Substantia nigra, reticulata	31 ± 2	22 ± 2^a^	−29	33 ± 2	28 ± 3	−15
Substantia nigra, compacta	37 ± 3	27 ± 1^a^	−28	39 ± 2	34 ± 3	−13
Limbic Areas						
Medial septal nucleus^b^	42 ± 2	29 ± 3^a^	−31	45 ± 3	38 ± 2	−16
Lateral septal nucleus	34 ± 3	22 ± 3^a^	−35	36 ± 4	26 ± 2^a^	−27
Bed nucleus of the stria terminalis	28 ± 3	17 ± 2^a^	−41	28 ± 2	20 ± 3^a^	−29
Basolateral amygdala	36 ± 3	22 ± 3^a^	−39	34 ± 3	30 ± 3	−12
Central amygdala	23 ± 3	17 ± 2	−26	25 ± 2	22 ± 3	−12

LCMRglc shown as mean ± SEM and % change in LCMRglu in DOI compared with saline-treated mice. *n* = 7. There was a main effect of 8-OH-DPAT treatment for all brain areas. CA, cornu ammonis; 5-HT1AR, serotonin 1A receptor; LCMRglc, local cerebral glucose utilization; UCN3OE, mice overexpressing urocortin 3.

a *p* < .05 versus saline.

b Main effect of genotype.

c Main effect of genotype × 8-OH-DPAT treatment.

**Table 2:** LCMRglc in Brain Regions of Control and UCN3OE Mice in Response to 5-HT2CR Agonist DOI

	Control			UCN3OE		
	Saline	DOI	%	Saline	DOI	%
Dorsal Raphé Nucleus	36 ± 3	37 ± 2	3	33 ± 5	30 ± 3	−9
Median Raphé Nucleus	45 ± 5	46 ± 3	2	45 ± 3	44 ± 3	−2
Neocortex						
Orbitofrontal^a^	58 ± 3	47 ± 2^b^	−19	63 ± 5	50 ± 3^b^	−21
Frontal^a^	43 ± 2	36 ± 1^b^	−16	45 ± 3	34 ± 3^b^	−24
Anterior cingulate^a^	46 ± 2	39 ± 2^b^	−16	48 ± 3	38 ± 2^b^	−21
Prefrontal^a^	44 ± 3	33 ± 3^b^	−20	47 ± 4	36 ± 2^b^	−23
Somatosensory	51 ± 4	47 ± 4	−8	55 ± 6	53 ± 3	−4
Parietal^a^	54 ± 3	42 ± 4^b^	−22	54 ± 5	40 ± 3^b^	−26
Posterior cingulate	49 ± 2	48 ± 2	−2	52 ± 4	52 ± 2	0
Piriform	40 ± 3	33 ± 2	−14	43 ± 3	39 ± 5	−10
Entorhinal	37 ± 3	32 ± 1	−13	35 ± 2	32 ± 3	−9
Hippocampus						
Molecular layer^a^	42 ± 3	34 ± 2^b^	−19	46 ± 3	35 ± 2^b^	−24
Dorsal subiculum	40 ± 3	44 ± 2	10	37 ± 3	36 ± 3	−3
Dentate gyrus^a^	26 ± 2	16 ± 2^b^	−38	24 ± 2	15 ± 1^b^	−38
Dorsal CA1^a^	37 ± 3	25 ± 3^b^	−32	38 ± 3	28 ± 2^b^	−26
CA2	35 ± 3	36 ± 4	3	32 ± 3	30 ± 3	−6
Ventral CA1	34 ± 2	30 ± 2	−13	31 ± 3	29 ± 2	−6
Ventral subiculum	30 ± 2	30 ± 4	0	33 ± 2	35 ± 3	6
CA3^a^	34 ± 2	24 ± 2^b^	−29	36 ± 2	25 ± 1^b^	−31
Extrapyramidal Areas						
Medial striatum^a^	41 ± 3	31 ± 2^b^	−24	42 ± 3	30 ± 2^b^	−22
Lateral striatum^a^	46 ± 3	29 ± 3^b^	−36	44 ± 3	31 ± 3^b^	−30
Globus pallidus	30 ± 2	32 ± 2	7	33 ± 2	32 ± 3	−3
Substantia nigra, reticulata	29 ± 2	30 ± 2	3	28 ± 2	28 ± 4	0
Substantia nigra, compacta	36 ± 2	37 ± 3	2	34 ± 3	34 ± 3	0
Limbic Areas						
Medial septal nucleus	40 ± 2	38 ± 3	−5	38 ± 3	38 ± 2	0
Lateral septal nucleus	34 ± 3	33 ± 3	−3	37 ± 4	36 ± 2	−2
Bed nucleus of the stria terminalis	27 ± 3	27 ± 2	0	25 ± 6	27 ± 3	8
Basolateral amygdala	33 ± 2	28 ± 4	−15	34 ± 5	30 ± 3	−12
Central amygdala	22 ± 2	18 ± 2	−18	25 ± 2	22 ± 3	−12

LCMRglc shown as mean ± SEM and % change in LCMRglu in DOI compared with saline-treated mice. *n* = 7. There was no main effect of genotype or interaction of genotype × DOI treatment for any brain area. CA, cornu ammonis; 5-HT2CR, serotonin 2C receptor; LCMRglc, local cerebral glucose utilization; UCN3OE, mice overexpressing urocortin 3.

a Main effect of DOI treatment.

b *p* < .05 versus saline.
