# Supplementary material for: Chronic Activation of Corticotropin-Releasing Factor Type 2 Receptors Reveals a Key Role for 5-HT1A Receptor Responsiveness in Mediating Behavioral and Serotonergic Responses to Stressful Challenge
Source: Biol Psychiatry. 2012 Sep 15;72(6):437–47. doi: 10.1016/j.biopsych.2012.05.005 (PMC3430862; doi:10.1016/j.biopsych.2012.05.005)
Supplement: Supplement 1 [file mmc1.pdf]

Chronic Activation of Corticotropin-Releasing Factor Type 2 Receptors Reveals a Key Role for Serotonin$_{1A}$ Receptor Responsiveness in Mediating Behavioral and Serotoninergic Responses to Stressful Challenge

Supplemental Information

Table S1. Details of tissue sample collection for high-performance liquid chromatography analysis of serotonin and 5-hydroxyindoleacetic acid concentrations.

Brain region	Rostrocaudal level (mm bregma)	Microdissections (number, diameter μm)
Lateral septal nucleus, intermediate part	0.80 to 0.50	4 (500)
Medial septum	0.80 to 0.50	2 (500)
Central amygdaloid nucleus, medial, lateral and capsular parts	-1.30 to -1.60	4 (410)
Basolateral amygdaloid nucleus, anterior part	-1.30 to -1.90	6 (410)
Subiculum	-3.70 to 4.00	6 (500)
Dorsal raphé nucleus, dorsal part	-4.30 to -4.60	2 (310)
Dorsal raphé nucleus, caudal part	-4.90 to -5.20	2 (310)

Table S2. CRF, CRFR1 and CRFR2 mRNA expression

	**Control**	**UCN3OE**
**CRF**		
Paraventricular Nucleus	1.00 ± 0.10	1.08 ± 0.20
Amygdala	1.00 ± 0.11	1.04 ± 0.03
Bed Nucleus of the Stria Terminalis	1.00 ± 0.11	1.03 ± 0.15
Hippocampus	1.00 ± 0.16	0.89 ± 0.11
**CRFR1**		
Paraventricular Nucleus	1.00 ± 0.06	1.09 ± 0.12
Amygdala	1.00 ± 0.07	1.09 ± 0.03
Bed Nucleus of the Stria Terminalis	1.00 ± 0.09	1.25 ± 0.10
Hippocampus	1.00 ± 0.06	1.22 ± 0.10
**CRFR2**		
Dorsal Raphé Nucleus	1.00 ± 0.07	1.08 ± 0.03
Lateral Septal Nucleus	1.00 ± 0.19	1.19 ± 0.17

mRNA, messenger RNA; CRF, corticotropin-releasing factor.
mRNA levels expressed as a ratio of control expression, mean ± SEM. n = 4-7.

Table S3. Table of *F*-values generated from 2-way analysis of variance of data from brain regions of control and UCN3OE mice treated with saline or 8-OH-DPAT. Critical $F_{(1,24)}$ value = 4.260 ($p < 0.05$).

	**Main Effect Genotype**	**Main Effect 8-OH-DPAT**	**Interaction Genotype x 8-OH-DPAT**
Dorsal Raphé Nucleus	1.588	12.020**	0.4808
Median Raphé Nucleus	2.380	13.540**	1.704
**Neocortex**			
Orbitofrontal	2.315	14.92**	2.315
Frontal	2.814	8.395**	1.140
Anterior Cingulate	0.379	8.015**	1.833
Prefrontal	7.407*	16.67**	7.407*
Somatosensory	0.333	7.053*	1.080
Parietal	0.136	9.470**	3.409
Posterior Cingulate	1.185	12.520**	2.667
Piriform	3.596	9.383**	1.723
Entorhinal	0.346	8.654**	1.885
**Hippocampus**			
Molecular Layer	5.921*	11.610**	2.132
Dorsal Subiculum	4.645*	4.645*	8.258**
Dentate Gyrus	0.900	16.901**	4.902*
Dorsal CA1	0.237	7.605*	1.289
CA2	2.632	6.737*	1.684
Ventral CA1	1.190	13.761**	3.857
Ventral Subiculum	0.615	5.538*	1.385
CA3	15.082**	37.232**	11.081**
**Extrapyramidal Areas**			
Medial Striatum	1.581	7.258*	0.807
Lateral Striatum	2.326	13.402**	4.558*
Globus Pallidus	3.556	10.890**	2.000
Substantia Nigra, reticulata	4.000	12.250**	1.000
Substantia Nigra, compacta	3.522	9.783**	1.087
**Limbic Areas**			
Medial Septal Nucleus	5.538*	15.380**	1.385
Lateral Septal Nucleus	0.947	12.741**	0.105
Bed Nucleus of the Stria Terminalis	0.346	24.042**	3.115

Basolateral Amygdala	1.000	9.000**	2.778
Central Amygdala	1.885	3.115	0.346

*$p < 0.05$.
**$p < 0.01$.

Table S4. Table of *F*-values generated from 2-way analysis of variance of data from brain regions of control and UCN3OE mice treated with saline or DOI. Critical $F_{(1,24)}$ value = 4.260 ($p < 0.05$).

	**Main Effect Genotype**	**Main Effect DOI**	**Interaction Genotype x DOI**
Dorsal Raphé Nucleus	2.128	0.085	0.304
Median Raphé Nucleus	0.077	0.000	0.077
Neocortex			
Orbitofrontal	1.362	12.260**	0.085
Frontal	0.000	14.090**	0.696
Anterior Cingulate	0.048	13.762**	0.429
Prefrontal	0.947	12.743**	0.000
Somatosensory	1.299	0.468	0.052
Parietal	0.068	11.461**	0.068
Posterior Cingulate	1.750	0.036	0.036
Piriform	1.723	2.574	0.192
Entorhinal	0.174	2.738	0.174
Hippocampus			
Molecular Layer	0.962	13.885**	0.346
Dorsal Subiculum	3.903	0.290	0.806
Dentate Gyrus	0.692	27.769**	0.077
Dorsal CA1	0.516	15.613**	0.129
CA2	1.884	0.023	0.209
Ventral CA1	0.762	1.714	0.190
Ventral Subiculum	1.939	0.121	0.122
CA3	0.692	33.923**	0.078
**Extrapyramidal areas**			
Medial Striatum	0.038	20.346**	0.346
Lateral Striatum	0.000	25.000**	0.444
Globus Pallidus	0.429	0.048	0.429
Substantia Nigra, reticulate	0.321	0.036	0.036
Substantia Nigra,compacta	0.807	0.032	0.032
**Limbic Areas**			
Medial Septal Nucleus	0.154	0.154	0.154
Lateral Septal Nucleus	0.947	0.105	0.000
Bed Nucleus of the Stria Terminalis	0.069	0.069	0.069

Basolateral Amygdala	0.167	1.500	0.019
Central Amygdala	2.333	2.333	0.0476

*$p < 0.05$.
**$p < 0.01$.

Table S5. Table of *F*-values generated from 2-way analysis of variance of 5-HT and 5-HIAA content of brain regions of control and UCN3OE mice under basal conditions or 24 hours post-stress. Critical $F_{(1,28)}$ value = 4.196 ($p < 0.05$).

	**Main Effect Genotype**	**Main Effect Stress**	**Interaction Genotype x Stress**
**5-HT**			
DRC	1.743	7.566**	0.815
DRD	0.891	8.477**	0.001
LSI	0.178	0.484	4.225*
MS	0.694	0.026	0.003
BLA	0.333	4.337*	0.083
CeA	2.464	0.515	0.027
S	0.242	3.166	0.777
**5-HIAA**			
DRC	0.553	13.278**	0.094
DRD	0.117	10.495**	0.631
LSI	1.383	0.15	5.371*
MS	1.022	3.471	0.000
BLA	0.186	0.187	0.143
CeA	1.841	1.432	0.007
S	0.216	2.037	0.011

5-HT, serotonin; 5-HIAA, 5-hydroxyindoleacetic acid; DRC, dorsal raphé nucleus, caudal part; DRD, dorsal raphé nucleus, dorsal part; LSI, lateral septum, intermediate part; MS, medial septum; BLA, basolateral amygdala; CeA, central amygdala; S, subiculum.
*p < 0.05.
**p < 0.01.

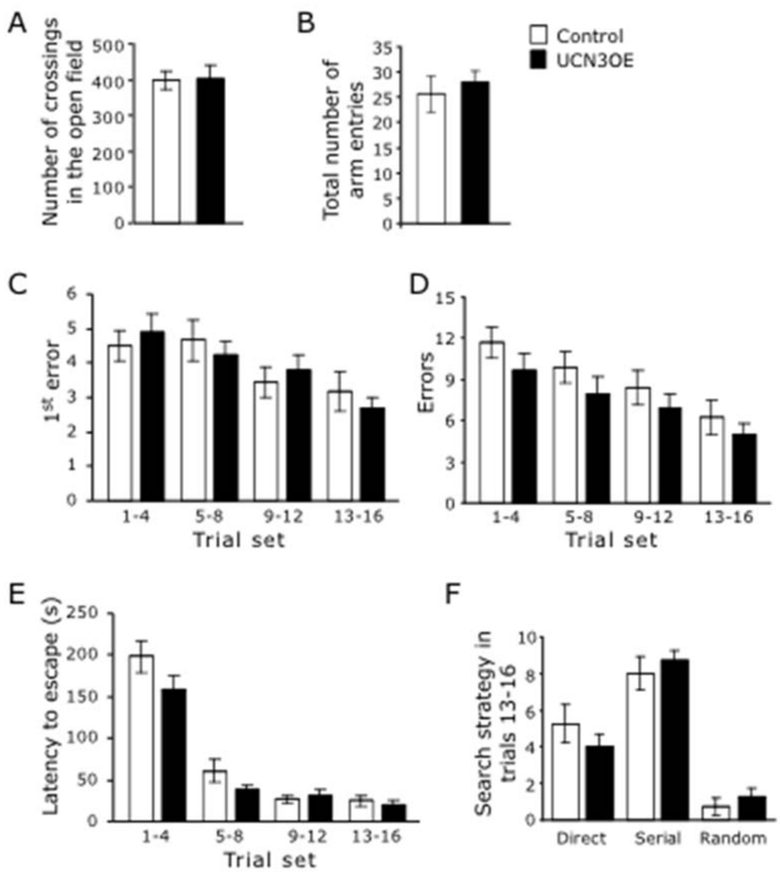

Figure S1. UCN3OE mice show similar locomotor activity and similar spatial learning to control mice. **(A)** Locomotor activity in the open-field test and **(B)** elevated-plus maze. *n* = 11-15. **(C)** Distance of the first hole searched from the escape hole (1st error), **(D)** total number of wrong holes searched, **(E)** latency to escape and **(F)** search strategy used during learning trials in the Barnes maze. *n* = 13-14.

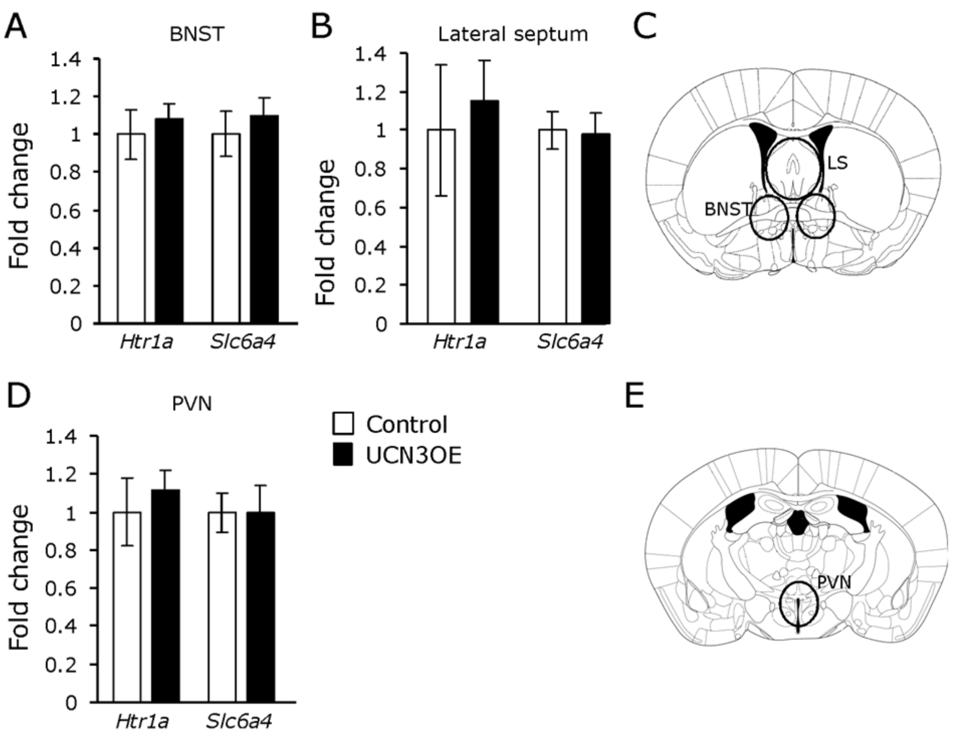

Figure S2. Basal *hrt1a* and *slc6a4* mRNA expression within stress-related brain regions in UCN3OE and control mice. BNST, bed nucleus of the stria terminalis; LS, lateral septum; PVN, paraventricular nucleus. Panels on the right depict the location of the tissue dissection. n = 4-7. (**C** and **E**) Reprinted from Paxinos G, Franklin KBJ (2001): The Mouse Brain in Stereotaxic Coordinates. 2nd ed. San Diego: Academic Press, with permission from Elsevier.
